# Oxaliplatin-Induced Peripheral Neuropathy via TRPA1 Stimulation in Mice Dorsal Root Ganglion Is Correlated with Aluminum Accumulation

**DOI:** 10.1371/journal.pone.0124875

**Published:** 2015-04-30

**Authors:** Jin-Hee Park, Jisook Chae, Kangsan Roh, Eui-Joon Kil, Minji Lee, Chung-Kyun Auh, Myung-Ah Lee, Chang-Hwan Yeom, Sukchan Lee

**Affiliations:** 1 Institute of Cancer Research, College of Medicine, The Catholic University of Korea, Seoul, Korea; 2 Department of Genetic Engineering, Sungkyunkwan University, Suwon, Korea; 3 The Institute of Life Science and Technology, Sungkyunkwan University, Suwon, Korea; 4 Department of Biological Science, Mokpo National University, Muan, Korea; 5 Department of Medical Oncology, Seoul St. Mary's Hospital, The Catholic University of Korea, Seoul, Korea; 6 Yeom’s Family Medicine Clinic, Seoul, Korea; Boston Children’s Hospital and Harvard Medical School, UNITED STATES

## Abstract

Oxaliplatin is a platinum-based anticancer drug used to treat metastatic colorectal, breast, and lung cancers. While oxaliplatin kills cancer cells effectively, it exhibits several side effects of varying severity. Neuropathic pain is commonly experienced during treatment with oxaliplatin. Patients describe symptoms of paresthesias or dysesthesias that are triggered by cold (acute neuropathy), or as abnormal sensory or motor function (chronic neuropathy). In particular, we found that aluminum levels were relatively high in some cancer patients suffering from neuropathic pain based on clinical observations. Based on these findings, we hypothesized that aluminum accumulation in the dorsal root ganglion (DRG) in the course of oxaliplatin treatment exacerbates neuropathic pain. In mice injected with oxaliplatin (three cycles of 3 mg/kg i.p. daily for 5 days, followed by 5 days of rest), we detected cold allodynia using the acetone test, but not heat hyperalgesia using a hot plate. However, co-treatment with aluminum chloride (AlCl_3∙_6H_2_O; 7 mg/kg i.p. for 14 days: equivalent 0.78 mg/kg of elemental Al) and oxaliplatin (1 cycle of 3 mg/kg i.p. daily for 5 days, followed by 5 days of rest) synergistically induced cold allodynia as well as increased TRPAl mRNA and protein expression. Inductively Coupled Plasma Mass Spectrometry (ICP-MS) analysis showed a significant increase in aluminum concentrations in the DRG of mice treated with aluminum chloride and oxaliplatin compared to aluminum chloride alone. Similarly, in a mouse induced-tumor model, aluminum concentrations were increased in DRG tissue and tumor cells after oxaliplatin treatment. Taken together, these findings suggest that aluminum accumulation in the DRG may exacerbate neuropathic pain in oxaliplatin-treated mice.

## Introduction

Oxaliplatin, a third-generation diaminocyclohexane (DACH) platinum drug, is widely used alone or in combination with fluorouracil and leucovorin to treat metastatic colorectal, ovarian, and pancreatic cancers [1–3]. However, oxaliplatin is associated with common and severe side effects. Within hours of oxaliplatin infusion, 90% of patients experience acute neuropathy characterized by paresthesias or dysesthesias triggered by exposure to cold. Likewise, chronic neuropathy develops after long-term treatment with oxaliplatin, leading to loss of sensory and motor function [4]. Oxaliplatin-induced peripheral neuropathy diminishes the quality of a patient’s life, resulting in dosage reductions and delays, and in some cases cessation of treatment [5]. Despite awareness of oxaliplatin-associated neuropathies and their severity, the underlying mechanisms are not well understood.

A useful marker for research into peripheral neuropathy, Transient Receptor Potential Ankyrin-1 (TRPA1), is activated by adversely cold temperatures [6]. TRPA1 localization is notable in sensory neurons of the dorsal root ganglia (DRG); however, TRPA1 is present not only on neuronal cells, but non-neuronal cells in the digestive system as well [7–10]. The DRG is located between the dorsal root and the spinal nerve and conveys sensory information from the peripheral to central nervous systems. Because of the lack of an efficient neurovascular barrier, high molecular weight compounds diffuse easily through the DRG [11]. This unique property predisposes the DRG to injury, which may initiate an increase in TRPA1 mRNA expression [12, 13]. Some authors have suggested that peripheral neuropathy is a neurological dysfunction induced by loss of afferent sensory neurons as a result of toxic substance accumulation in the DRG [12–14].

Aluminum (Al) is the third most abundant element in the earth’s crust and is present in our food as well as certain sources of drinking water and some medicines. To varying degrees, green plants accumulate Al along with other minerals from the soil. Al is not an essential element in the human diet, and bioaccumulation of Al is related to neurotoxicity and pathological conditions such as dialysis encephalopathy and osteomalacia [15–17]. Al induces organ toxicities affecting the kidneys, bones, brain, blood, and nervous system [18]. Despite increasing awareness that environmental Al exposures may lead to neurotoxicity, the mechanisms responsible for Al toxicity remain undefined. Likewise, efforts to date aimed at improving peripheral neuropathies from various causes have had unsatisfactory results.

Based on clinical observations of chemo-induced neuropathy, we hypothesized that bioaccumulation of aluminum may be associated with several aspects of neurotoxicity, and that TRPA1 activation facilitates induction of cold hyperalgesia and allodynia.

The purpose of this study was to reveal the synergistic effect of Al accumulation on oxaliplatin-induced peripheral neuropathy by activation of TRPA1. For this investigation, mice were treated with oxaliplatin and/or aluminum chloride, and subsequent induction of cold hyperalgesia or allodynia was analyzed by behavioral tests. Accumulation of metals in the DRG was analyzed by inductively coupled plasma mass spectrometry (ICP-MS), and TRPA1 expression was assessed by immunological staining and real-time PCR.

## Materials and Methods

### Cancer cell line and culture conditions

CT26 mouse colon carcinoma cells, which were used for the tumor-induced model, were obtained from ATCC (American Type Culture Collection, Manassas, VA, USA). Cells were maintained in Dulbecco’s modified Eagle’s medium (DMEM; Hyclone, Logan, UT, USA) at 37°C in a humidified atmosphere containing 5% CO_2_. Medium was supplemented with 10% fetal bovine serum (FBS; Hyclone) and 100 U/ml penicillin-streptomycin (Hyclone).

### Experimental animals

For our *in vivo* study, ICR and BALB/c male mice were obtained at 4 weeks of age from DBL Co., Ltd (Daehan Bio Link, Eumseong, Korea). All mice weighed 27–29 g (ICR mice) or 20–21 g (BALB/c mice) and were allowed a one-week acclimation period. The animals were maintained under a normal light/dark cycle at 22 ± 1°C and given a gamma ray-sterilized normal diet (Teklad Global 18% Protein Rodent Diet; Harlan Laboratories Inc., IN, USA) and autoclaved tap water. The components of the mouse diet are listed in Table 1. This study was carried out in strict accordance with the recommendations in the Guide for the Care and Use of Laboratory Animals of the National Institutes of Health. The protocol was approved by the Institutional Animal Care and Use Committee (IACUC) of the Catholic University in Korea (Permit number: CUMC-2012-0161-01). All behavioral experiments were conducted in the same room and in a randomized order before and after drug treatment.

**Table 1:** Essential nutrient and non-nutrient mineral components of the mouse diet.

Non-nutrient minerals	μg/g	Essential minerals	mg/g
Hg	0.00	Na	2.4651
Pb	0.07	K	7.4180
Al	52.22	Ca	10.9815
Ba	9.91	Mg	3.4912
Cd	0.00	Zn	0.1416
As	0.21	S	3.0638
U	0.44	P	7.51
Bi	0.00	Mn	0.1357
Tl	0.00	Fe	0.3211
Cs	0.04	Cu	0.1091
Pt	0.01	Se	0.0004

### Drug treatment regimens

**Fig 1:**
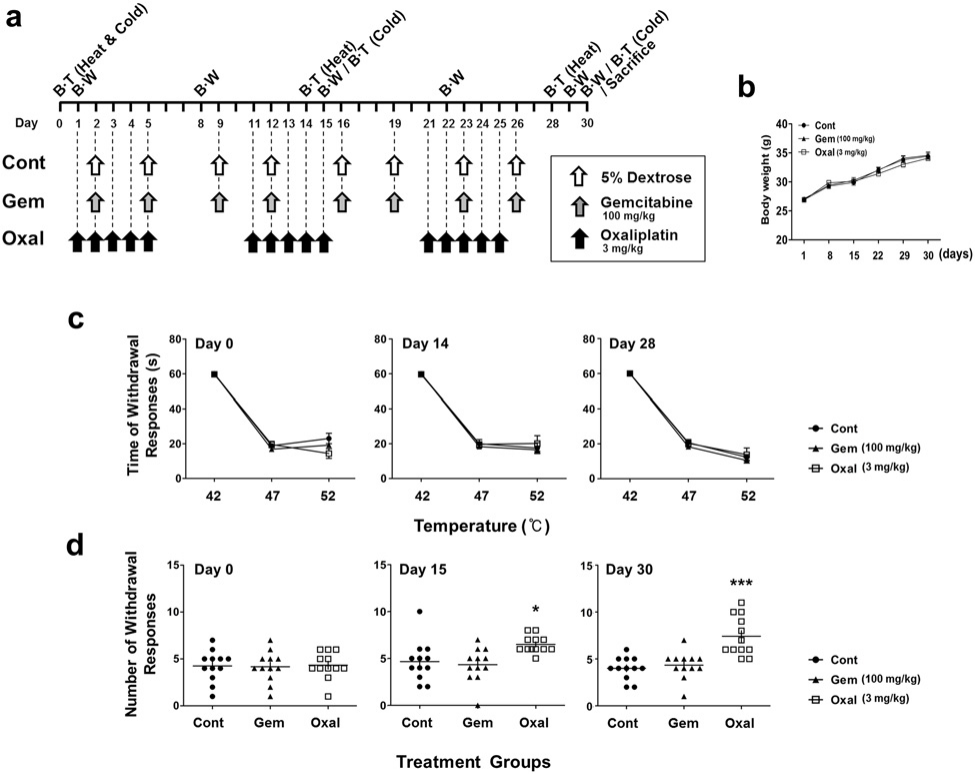
Peripheral neuropathy induced by platinum-based oxaliplatin. 5% dextrose (Cont), gemcitabine (100 mg/kg; Gem), and oxaliplatin (3 mg/kg; Oxal) were administered by i.p. injection for 30 days as shown in the schedule (a). Body weight was measured at 7-day intervals from the initial treatment (b). Hot plate test for thermal hyperalgesia was performed before the first infusion and again every 14 days. There was no significant difference between control (●) and drug-treated (▲: gemcitabine, □: oxaliplatin) mice (c). Acetone test for cold allodynia was performed before the first infusion and repeated every 15 days. On day 30, paw withdrawal responses to cold stimuli were significantly increased in only the Oxal group (d). Results are representative of two independent experiments. Values are expressed as the mean ± SEM (*n* = 12 per group). *p < 0.05, ***p < 0.001 compared with the control group. BT: behavioral test, BW: body weight

**Fig 2:**
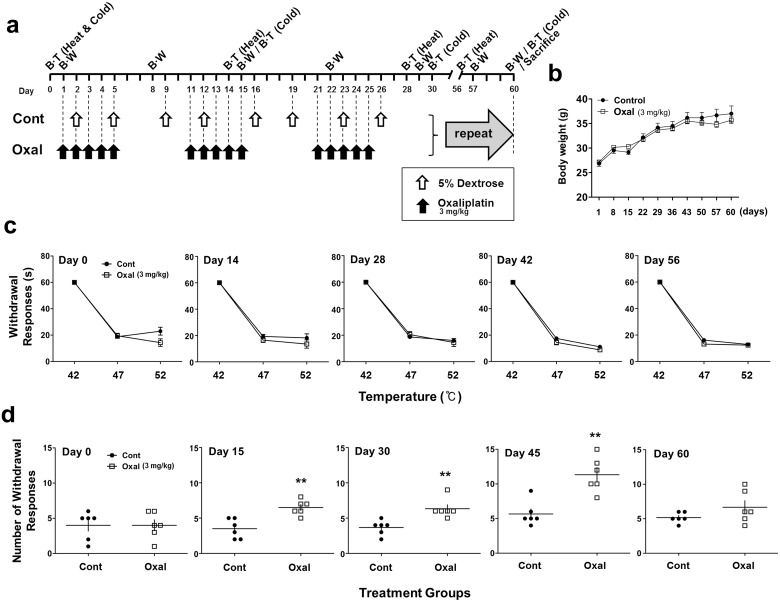
Induction of peripheral neuropathy after long-term exposure to oxaliplatin. 5% dextrose (Cont) and oxaliplatin (3 mg/kg; Oxal) were administered by i.p. injection for 60 days as shown in the schedule (a). Body weight was measured every 7 days from the initial treatment (b). Hot plate test for thermal hyperalgesia was performed before the first infusion and again every 14 days. There were no significant differences between control (●) and drug-treated (□: oxaliplatin) mice (c). Acetone test for cold allodynia was performed before the first infusion and again every 15 days. Paw withdrawal responses for cold stimuli showed a sudden decrease on day 60 after a peak on day 45 (d). Results are representative of three independent experiments. Values are expressed as the mean ± SEM (*n* = 6 per group). **p < 0.01 compared with the control group. BT: behavioral test, BW: body weight

**Fig 3:**
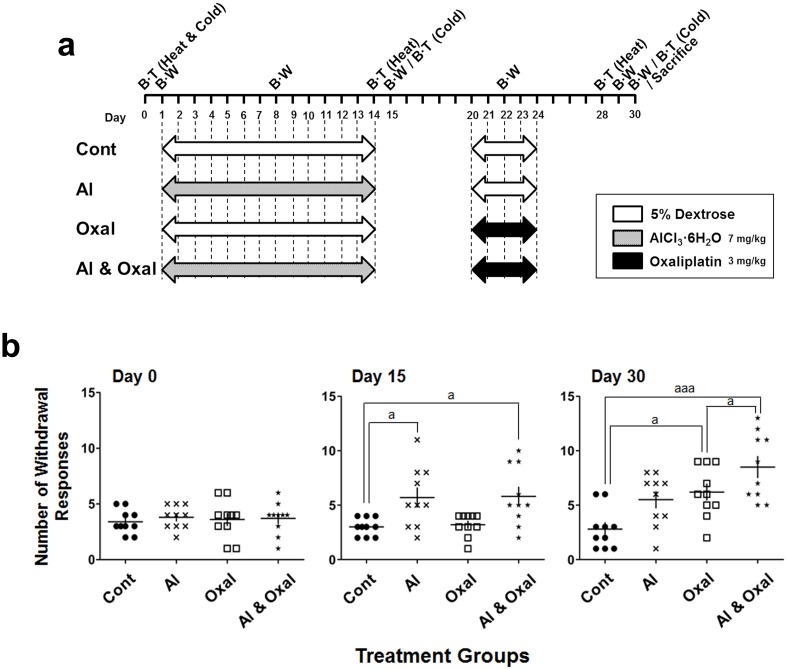
Exacerbation of peripheral neuropathy induced by combined treatment with oxaliplatin and aluminum chloride. 5% dextrose, aluminum chloride (AlCl_3_·6H_2_O, 7 mg/kg; equivalent 0.78 mg/kg of elemental Al), and oxaliplatin (3 mg/kg) were administered by i.p injection for 24 days including 5 days of rest. (a). Acetone test for cold allodynia was performed before the first infusion and then every 15 days (b). Hypersensitivity to cold stimuli was synergistically increased in mice treated with both oxaliplatin and aluminum chloride. Cont (●: 5% dextrose), Al (x: aluminum chloride, 7 mg/kg), Oxal (□: oxaliplatin, 3 mg/kg), Al & Oxal (★: aluminum chloride 7 mg/kg and oxaliplatin 3 mg/kg). Results are representative of two independent experiments. Values are expressed as the mean ± SEM (*n* = 10 per group). ^a^ *p* < 0.05, ^aaa^ *p* < 0.001 compared with the control or oxaliplatin group. BT: behavioral test, BW: body weight

Mice used for *in vivo* experiments underwent baseline measurements (cold or hot plate test) of pain sensitivity before beginning treatment. The doses and dosing schedules of anticancer drugs corresponded to patient treatment regimens such as amounts and cycles, and were modified for the mouse model. All administrations were performed by i.p injection at defined time intervals (Figs 1a, 2a, and 3a).

#### Short-term (acute)

In the first set of experiments, we randomized subjects into three treatment groups including those that received gemcitabine (100 mg/kg; Boryung Co. Ltd., Seoul, Korea), oxaliplatin (3 mg/kg; Boryung Co. Ltd., Seoul, Korea), or 5% dextrose (Joongwae Pharmaceutical, Seoul, Korea). Gemcitabine is an anticancer drug that does not induce peripheral neuropathy, and was used as a positive control. We used 5% dextrose as a vehicle for preparing oxaliplatin and gemcitabine, which are water-soluble agents, and doses were based on previous reports [19, 20]. A first group of mice was treated with daily intraperitoneal (i.p) injections of oxaliplatin for 5 days, followed by 5 days of rest during three cycles (Oxal). A second group was treated with i.p injection of gemcitabine twice weekly with 2 or 3 days of rest between injections during four cycles (Gem). The control group was treated with i.p injection of 5% dextrose according to the same schedule as gemcitabine-treated group (Cont). After treatments were initiated, behavioral tests including paw thermal hyperalgesia (hot plate test; every 14 days) and paw cold allodynia (acetone test; every 15 days) were conducted (*n* = 12 per group, Fig 1A). Two independent experiments were performed.

#### Long-term (sub-acute)

In a second set of experiment for long-term treatment, we randomized subjects into two treatment groups consisting of 5% dextrose or oxaliplatin (3 mg/kg). Specifically, the control group was treated with i.p injection of 5% dextrose twice weekly with 2 or 3 days of rest between injections, with a total of eight cycles (Cont). Another group was treated with i.p injection of oxaliplatin for 5 days, followed by 5 days of rest for a total of six cycles (Oxal). Behavioral tests including the hot plate test (every 14 days) and the acetone test (every 15 days), and were conducted after beginning treatments (*n* = 6 per group, Fig 2a). Three independent experiments were performed.

#### Combinational treatment of aluminum and oxaliplatin

In a third set of experiments, mice were randomized into four groups. The first group of mice was treated with 5% dextrose for 14 days followed by 5 days, with an interval of 5 days between the two treatments (Cont). In the second group, after 14 days of aluminum chloride (AlCl_3∙_6H_2_O, 7 mg/kg; Sigma-Aldrich, St. Louis, MO, USA) treatment, 5% dextrose was administered for 5 days, followed by 5 days of rest (Al). A third group was treated with oxaliplatin (3 mg/kg) for 5 days after administering 5% dextrose followed by 5 days of rest (Oxal). In the last group, mice were administrated aluminum chloride mice for 14 days prior to treatment with oxaliplatin for 5 days followed by 5 days of rest (Al & Oxal). The dose of aluminum chloride was based on the report of Guo *et al*., and is equivalent to 0.78 mg/kg of elemental Al [21]. This dose of elemental Al is lower than the ingested amounts (12–71 mg Al/kg/day) by an aluminum containing medication such as antacid/anti-ulcer products and higher than the amount of daily aluminum intake (0.10–0.12 mg Al/kg/day for adult males and females) from the diet, when compare to human exposure [22]. The acetone test was subsequently conducted every 15 days [19, 23] (*n* = 10 per group, Fig 3a). Two independent experiments were performed.

### Tumor-induced murine model & treatments

To test the correlation between metal accumulation and oxaliplatin treatment in a murine induced-tumor model, we used five-week-old male BALB/c mice (*n* = 10 per group). Each mouse was subcutaneously inoculated on the back of the neck with CT26 mouse colon cancer cells (10^6^ cells/mouse). Mice were treated with 5% dextrose (Tumor Induced Model: TIM) or oxaliplatin (TIM & Oxal; 3 mg/kg) from day 14 after transplantation for 5 days. At day 7 after the final infusion, all mice were sacrificed as described below. The DRG and tumor tissues formed on the back were subsequently extracted for ICP-MS (Inductively coupled plasma mass spectrometry) analysis [24]. Two independent experiments were performed.

### Assessment of general toxicity

Body weights (g) were recorded every 7 days, including on the day of treatment and immediately prior to sacrifice. Mice were examined daily for abnormal clinical signs such as piloerection, hindlimb weakness, gait disturbance or gastrointestinal disorders such as diarrhea.

### Behavioral testing

Behavioral testing was conducted by an observer who was blinded to the treatment grouping. Animals were habituated to the testing environment for more than 30 min prior to the start of each test.

#### Paw thermal hyperalgesia: hot plate test

The hot plate test was used to evaluate the effects of oxaliplatin and gemcitabine on sensory neuropathy. Animals were placed on heated plates at 42, 47, and 52 ± 0.2°C until the first episode of jumping or hind paw licking, or for a maximum of 60 seconds. The hot plate test was conducted before the first treatment and again every 14 days regardless of the treatment cycle, and was repeated at least three times at 10-min intervals. Latencies were expressed as the average of results. If a response was not observed, the 60 s cut-off time was recorded [19, 25].

#### Cold allodynia of the paw: acetone test

Subjects were placed on a wire mesh floor and a drop (0.05 ml) of acetone was applied to one of the hind paws. Responses were monitored for 20 seconds after the application. If the subject did not withdraw, flick, or stamp the paw within 20 seconds, zero points were recorded for the trial. However, if the animal responded to the cooling effect of the acetone during the first 20 seconds, the response was assessed for a total of 40 seconds from initial application. Responses to acetone were graded according to the following 4-point scale: 0, no response; 1, quick withdrawal, flick or stamp of the paw; 2, prolonged withdrawal or repeated flicking (≥ 2) of the paw; and 3, repeated flicking of the paw with licking directed at the paw. Acetone testing was conducted before the first infusion and again every 15 days regardless of the treatment cycle. The stimulus was applied alternately three times to each paw and the responses were scored categorically. Cumulative scores were obtained by averaging the six scores for each mouse [26, 27].

### Isolation of DRG neurons

After all treatments were completed, mice were intraperitoneally injected with a cocktail of tiletamine/zolazepam (100 mg/kg) and xylazine (10 mg/kg), and subsequently sacrificed with an excess of anesthetic. We dissected the DRG segments of T_1_—T_12_ and L_1_—L_5_ on the spine of a mouse, and the procedures were performed as described by Saijilafu *et al*.[28].

### Analysis of metal concentration

The analysis of metal concentration in the DRG and tumor tissues was carried out by the Korea Research Institute of Analytical Technology (Daejeon, Korea). DRG and tumor tissues were dissected from each animal, and the tissues were frozen in liquid nitrogen and stored at -80°C. To analyze non-essential minerals (metals) and minerals in the diet, we prepared 2 g of frozen mouse chow by grinding it into a powder in liquid nitrogen followed by analytical determination of tissue metal concentration using Inductively Coupled Plasma Mass Spectrometry (ICP-MS) on a Thermo Elemental-X7 (Thermo Electron, London, UK) [24].

### Real-time polymerase chain reaction (PCR)

Total RNA was isolated from DRG tissue using a tissue homogenizer and TRIzol Reagent (Invitrogen, Carlsbad, CA, USA) according to the manufacturer's instructions. RNA from each sample (1 μg) was reverse transcribed into cDNA using oligo-dT primers and Moloney Murine Leukemia Virus (MMLV) reverse transcriptase (Superbio Co., Daejon, Korea). Quantitative real-time PCR was performed in triplicate using SYBR *Premix Ex Taq* (TAKARA Bio Inc., Shiga, Japan). The primer sequences were as follows: TRPA1, 5'-CCATGACCTG-GCAGAATACC-3' (forward) and 5'-TGGAGAGCGTCCTTCAGAAT-3' (reverse); GAPDH, 5'-ACCCAGAAGACTGTGGATGG-3' (forward) and 5'-CACATTGGGGGTAGGAACAC-3' (reverse). The reaction conditions were as follows: reverse transcription at 55°C for 30 minutes; initial PCR activation at 94°C for 15 minutes; and 40 cycles of denaturing, annealing, and extension at 94°C for 15 seconds, 55°C for 30 seconds, and 72°C for 30 seconds, respectively. The sizes of the amplified products were confirmed by gel electrophoresis. The level of each transcript was expressed as the ratio of TRPA1 to GAPDH mRNA. The normalized value from the control group was arbitrarily assigned a value of 1.

### Immunofluorescence analysis of the DRG

For immunofluorescence staining, the extracted DRG was fixed in 4% (w/v) paraformaldehyde at 4°C overnight. The samples were then embedded in OCT compound (Sakura Finetek Japan Co. Ltd., Tokyo, Japan) and frozen at -80°C. DRG sections (7 μm thick) were prepared on gelatin-coated glass slides using a cryocut microtome (Leica CM3050S, Wetzlar, Germany). The slides were blocked with 5% fetal bovine serum (HyClone) and 2% bovine serum albumin (MP Biomedicals, Santa Ana, CA, USA) in PBS for one hour at room temperature (RT), followed by incubation with rabbit anti-TRPA1 (1:200; Abcam plc., Cambridge, UK) at 4°C overnight. The tetramethylrhodamine-5-(and-6)-isothiocyanate (TRITC)-conjugated anti-rabbit secondary antibody (1:500; Vector Laboratories Inc., Burlingame, CA, USA) was incubated for one hour prior to visualization. The sections were mounted with Vectashield mounting medium (Vector Laboratories), and fluorescent images were obtained using a confocal microscope (LSM 5 Pascal, Carl Zeiss, Germany).

### TUNEL assay for cell death

Cell death in DRG tissue was evaluated using an *in situ* cell death detection kit (Roche Applied Science, PA, USA) based on terminal deoxynucleotidyl transferase-mediated dUTP nick end labeling (TUNEL) staining. Frozen-sectioned slides were stained with the TUNEL mixture according to the manufacturer’s instructions after applying microwave irradiation in 0.1 M citrate buffer (pH 6.0) for one minute. Stained slides were mounted using Vectashield mounting medium with 4',6-diamidino-2-phenylindole (DAPI) and analyzed by confocal microscopy (BX51; Olympus Corp., Tokyo, Japan) [29]. Images were monitored by means of fluorescence microscope Axiovert 200 (ZEISS, Oberkochen, Germany) and the number of TUNEL-positive cells within each image was analyzed by using AxioVision Image Analysis.

### Statistical analysis

All data were expressed as the mean ± standard error of the mean (SEM). Graphing and analysis of results was performed using Graph Pad Prism version 5 (Graphpad Software, San Diego, CA, USA). One-way or two-way ANOVA was performed to test differences in responses across treatment groups. The statistical significance of changes in mRNA levels was tested using the Student’s *t*-test. A *p*-value < 0.05 was considered to be statistically significant. In behavior tests and TUNEL assay, post-hoc comparisons for experimental groups were performed with Turkey’s honestly significant difference test.

## Results

### General toxicity of oxaliplatin and gemcitabine

Mice were injected with either oxaliplatin (3 mg/kg) or gemcitabine (100 mg/kg) at dosages corresponding to human chemotherapy, while 5% dextrose was used as a control treatment (Fig 1a). No deterioration in general status was observed in any of the groups, and no mice died in the course of our experiments. No significant differences in body weight were observed between groups at any time (Figs 1b and 2b).

### Thermal hyperalgesia and cold allodynia responses in an acute oxaliplatin model

To assess peripheral neuropathy induced by the treatment regimen used in this mouse model, we conducted behavioral tests for thermal hyperalgesia and cold allodynia. The schedule of drug treatments for the acute model is shown in Fig 1a. No significant differences were observed between treatment and vehicle groups during the hot plate test for thermal hyperalgesia at 42°C, 47°C, and 52°C at day 28 (Fig 1c). In addition, there was no significant difference between groups with respect to the number of withdrawal responses in the acetone test for cold allodynia on the day before treatment. However, the oxaliplatin-treated group exhibited an increase in withdrawal responses after 15 days, and a significant increase in withdrawal responses on day 30 (*p* < 0.001) after the start of treatment (Fig 1d). As shown in Fig 1c and 1d, gemcitabine treatment did not affect the responses to either hot or cold stimuli.

### Thermal hyperalgesia and cold allodynia responses in a sub-acute oxaliplatin model

The schedule of treatments for the sub-acute model is shown in Fig 2a. No significant differences were observed between groups during the hot plate test for thermal hyperalgesia at 42°C, 47°C, and 52°C at day 56 after drug or 5% dextrose treatment (Fig 2c). In the results of acetone test for cold allodynia, the withdrawal response increased from day 15 (*p* < 0.05) to day 45 after treatment (*p* < 0.001). However, the cold responses were decreased at day 60 after treatment, at which time the oxaliplatin group and the control group (Fig 2d) did not differ significantly in either the thermal hyperalgesia or cold allodynia test results.

### Effects of oxaliplatin and aluminum chloride combination treatment on cold-evoked peripheral neuropathy

To test the effects of the combination of oxaliplatin and aluminum chloride on peripheral neuropathy, we conducted the acetone test according to a schedule shown in Fig 3a. On the day treatment was initiated (day 0), the groups did not differ significantly in the number of withdrawal responses. After 14 days of treatment prior to the acetone test for cold allodynia, the numbers of withdrawal responses in the groups treated with either aluminum chloride alone or in combination with oxaliplatin (Al and Al & Oxal) was significantly increased (day 15, *p* < 0.05). On day 30 (6 days after the end of oxaliplatin treatment), all oxaliplatin-treated groups exhibited increases in the number of withdrawal responses (Fig 3b). Combination treatment with oxaliplatin and aluminum chloride induced cold-evoked peripheral neuropathy of a greater intensity than did treatment with 5% dextrose (Cont) or aluminum chloride alone (*p* < 0.001). The number of withdrawal responses did not change between day 15 (*p* < 0.05) and day 30 (*p* < 0.05) in the group treated with aluminum chloride alone. As shown in Figs 1d and 2d, oxaliplatin treatment resulted in cold allodynia.

### Accumulation of Al and Pt in DRG tissue of oxaliplatin-treated mice

**Fig 4:**
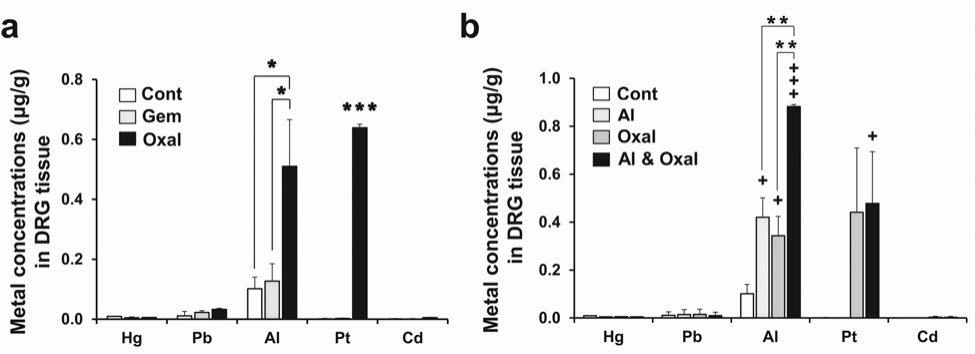
Accumulation of aluminum (Al) and Platinum (Pt) in DRG tissue after drug treatment. Total elemental contents including Al and Pt were assayed in DRG tissues by inductively coupled plasma mass spectrometry (ICP-MS) after performing treatments for 30 days according as the schedules shown in Figs 1a (a) and 3a (b). Al accumulated to significantly higher concentrations in the DRG from the Oxal (oxaliplatin 3 mg/kg) group than in the DRG from Cont (5% dextrose) or Gem (gemcitabine 100 mg/kg) groups (*n* = 6 per group) (a). Effect of combined treatment with oxaliplatin and aluminum chloride on the elemental composition of DRG tissues. The DRGs from the Cont (5% dextrose), Al (aluminum chloride 7 mg/kg), Oxal (oxaliplatin 3 mg/kg), Al & Oxal (aluminum chloride 7 mg/kg; equivalent 0.78 mg/kg of elemental Al, oxaliplatin 3 mg/kg) groups were analyzed by ICP-MS. Al concentrations increased synergistically in tissues of the combined-treatment group compared to the Al or Oxal groups (*n* = 10 per group) (b). Results are representative of two independent experiments. Values are expressed as the mean ± SEM. *p < 0.05, ***p < 0.001 compared with the Gem group, ^+^ *p* < 0.05, ^+++^ *p* < 0.001 compared with the Cont group, **p < 0.01 compared with Oxal and Al & Oxal.

The concentrations of Al in the DRG of oxaliplatin-treated mice were five times higher than those of the Cont group (*p* < 0.05, Fig 4a). Heavy metals such as Hg, Pb and Cd were not significantly elevated in any of the groups. Significant accumulation of Pt in the DRG was observed only in the Oxal group, as it is a Pt-based anticancer drug (*p* < 0.05). The Gem group showed elevated Al levels in DRG (0.13 μg/g), although these were lower than in the Oxal group (Fig 4a). The metal and mineral concentrations in the food provided during the experiments are listed in Table 1. All non-essential metals and minerals except for Al were present in the diet at concentrations ranging from 0.01 μg/g to 10 μg/g and from 0.4 μg/g to 11 mg/g, respectively. However, mouse chow contained Al at a much higher concentration (52.2 μg/g) than the other metals and minerals. Although all mice were fed the same diet and did not exhibit significant differences in total food intake (data not shown), Al concentrations in DRG tissues from the Oxal group were 4.2-fold higher than in the Gem group and 5-fold higher than in Cont group (*p* < 0.05). Concentrations of Mg, Mn and Se in the DRG differed from control values in both the groups treated with gemcitabine- and oxaliplatin alone. In addition, the levels of P were significantly decreased in both groups (p < 0.05; Table 2).

**Table 2:** Content of essential minerals in mouse DRG tissues (μg/g) after chemotherapy.

	Treatment Groups (n = 6 per group)		
Minerals	Control^a^	Gem^b^	Oxal^c^
	(5% Dextrose)	(100 mg/kg)	(3 mg/kg)
Na	3245.27 ± 332.32	3800.14 ± 31.86	3185.47 ± 232.29
K	566.57 ± 128.56	545.17 ± 64.22	537.22 ± 12.02
Ca	236.77 ± 225.19	156.71 ± 65.37	491.49 ± 390.72
Mg	70.16 ± 10.94	46.39 ± 2.92 *	51.33 ± 7.46
Zn	5.96 ± 0.02	6.09 ± 1.45	13.57 ± 12.39
S	697.47 ± 86.53	639.52 ± 48.91	634.49 ± 43.01
P	2545.32 ± 175.02	2049.46 ± 77.29 *	2028.76 ± 34.77 *
Mn	0.22 ± 0.04	0.40 ± 0.06 *	0.33 ± 0.09
Fe	12.23 ± 0.074	12.33 ± 1.74	10.33 ± 1.50
Cu	1.96 ± 2.78	2.93 ± 0.73	3.26 ± 0.76
Se	0.54 ± 0.04	0.65 ± 0.15	0.40 ± 0.05 *

^a^ control: 5% dextrose.

^b^ gemcitabine: 8 intraperitoneal infusions per mouse over 30 days.

^c^ oxaliplatin: 15 intraperitoneal infusions per mouse over 30 days.

* *p* < 0.05 compared to control.

### Effects of treatment with oxaliplatin and aluminum chloride combined on metal accumulation in DRG tissue

Combined treatment with oxaliplatin and aluminum chloride (Al & Oxal) resulted in significantly higher concentrations of Al in DRG tissue compared with groups treated individually with 5% dextrose (Cont), oxaliplatin (Oxal), or aluminum chloride (Al) (Fig 4b). Although the accumulation of Al within the Oxal group exceeded control values, the combination treatment resulted in a more than 8.5-fold increase compared with the Cont group. Pt accumulation was also detected in DRG tissues of the Oxal and Al & Oxal groups.

### Accumulation of Al in DRG tissue and tumor cells in a murine induced-tumor model after oxaliplatin treatment

**Fig 5:**
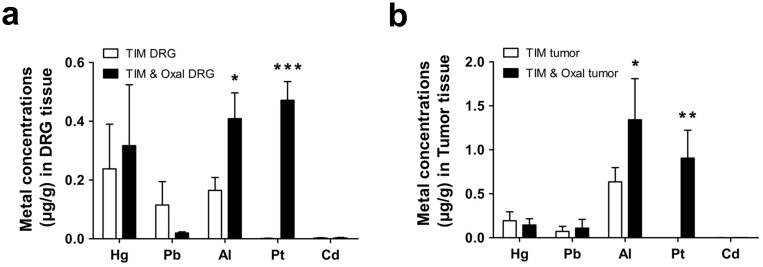
Al accumulation in DRG and tumor tissue of oxaliplatin-treated mice with induced tumors. Tumors were induced in mice by injection of murine CT26 colon cancer cells. 5% dextrose (Tumor Induced Model: TIM) and oxaliplatin (TIM & Oxal; 3 mg/kg) were administered by i.p. injection on days 14 through 18 after transplantation. The extracted DRG and tumor tissues were analyzed by inductively coupled plasma mass spectrometry (ICP-MS). Figures show the increases in Al and Pt concentrations in DRG (a) and tumor (b) tissues from oxaliplatin-treated mice. Results are representative of two independent experiments. Values are expressed as the mean ± SEM (*n* = 10 per group). *p < 0.05, **p < 0.01, and ***p < 0.001 compared with the control group.

ICP-MS revealed higher levels of Al accumulation in the DRG of the oxaliplatin-treated mice with induced tumors (TIM & Oxal) than in mice with induced tumors that were not treated (TIM; Fig 5a). Increased Al accumulation was also observed in non-tumor-bearing mice treated with oxaliplatin (Fig 4b). In comparison with tumor-deficient and tumor-bearing mice, the concentration of Al was slightly increased in the DRGs of tumor-bearing mice (TIM: 0.17 ± 0.05 μg/g, TIM & Oxal: 0.41 ± 0.11 μg/g) than in tumor-deficient mice (Cont: 0.1 ± 0.04 μg/g, Oxal: 0.34 ± 0.08 μg/g) (Figs 4b and 5a). Pt accumulation was confirmed in both DRG and tumor tissues which were treated with oxaliplatin, as it is a Pt-based anticancer drug (Fig 5). Moreover, Al accumulation in tumor tissues of the TIM group exceeded the levels in DRG of the same groups and the highest levels of Al accumulation was measured in tumor tissues of the TIM & Oxal group (Fig 5b).

### Effects of oxaliplatin treatment on TRPA1 protein and mRNA levels in the acute model

**Fig 6:**
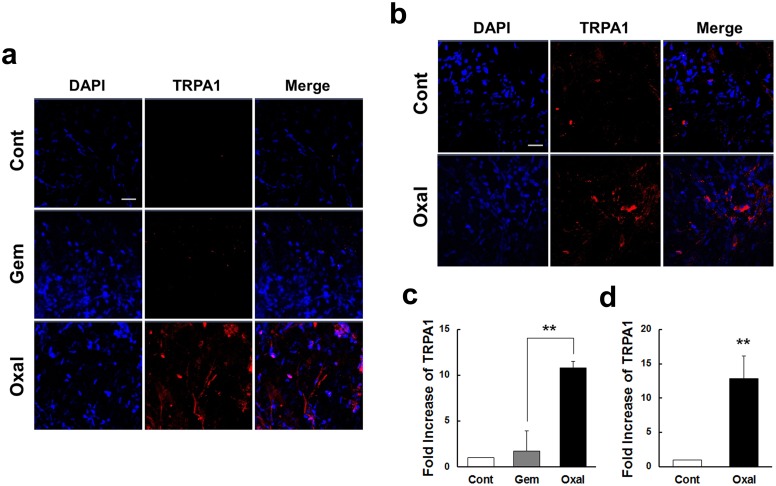
TRPA1 mRNA and protein expression in DRG from mice after short-term (30 days) (a, c) and long-term (60 days) (b, d) exposure to oxaliplatin. DRG tissues from Cont (5% dextrose), Gem (gemcitabine, 100 mg/kg), and Oxal (oxaliplatin, 3 mg/kg) groups were harvested at day 4 or 5 after final infusion with reagents. (a, b) Immunofluorescent staining for protein expression was conducted on whole DRG tissues with anti-TRPA1 (red). Nuclei were stained with DAPI (blue) and visualized using a confocal scanning microscope. TRPA1 protein was increased significantly in the Oxal group. (c, d) The relative ratio of TRPA1 mRNA measured by quantitative real-time PCR increased significantly in the Oxal group compared with tissues from the Gem (c) and Cont (c, d) groups. TRPA1 levels are expressed as fold changes after normalizing to 28S RNA. Assays were conducted in triplicate, and results are representative of three independent experiments. Values are expressed as the mean ± SEM (*n* = 6 per group). **p < 0.01 compared with the gemcitabine or control group. Scale bar = 10 μm for all panels.

Changes in TRPA1 protein expression after acute oxaliplatin treatment were assessed using confocal microscopy following immunofluorescent staining of whole DRG sections with an anti-TRPA1 antibody. A very weak signal was observed in DRG tissues from the Cont and Gem groups of mice. The DRG tissues from the Oxal group, however, displayed significant signals as compared to the Cont and Gem groups (Fig 6a). We evaluated the mRNA expression of TRPA1 in DRG tissues harvested from 5% dextrose, oxaliplatin and gemcitabine-treated mice after treatment for 30 days (*p* < 0.01, Fig 6c). Based on quantitative real-time PCR assay, TRPA1 expression increased more than 10-fold in the Oxal group compared to the Cont group. However, TRPA1 expression in the DRG of the Gem group was not significantly increased.

### Effects of oxaliplatin treatment on TRPA1 protein and mRNA levels in a sub-acute model

To evaluate the protein and mRNA expression of TRPA1, we harvested DRG tissues from 5% dextrose and oxaliplatin-treated mice after long-term sub-acute treatment (60 days: *p* < 0.01, Fig 6b and 6d). The expression of TRPA1 protein in the sub-acute model also showed strong signals in whole DRG tissues from the Oxal group compared with the Cont group (Fig 6b). With respect to mRNA expression, the Oxal group exhibited increased levels of TRPA1, more than 12-fold compared to the Cont group (Fig 6d).

### Effects of combined treatment with oxaliplatin and aluminum chloride on TRPA1 protein and mRNA expression

**Fig 7:**
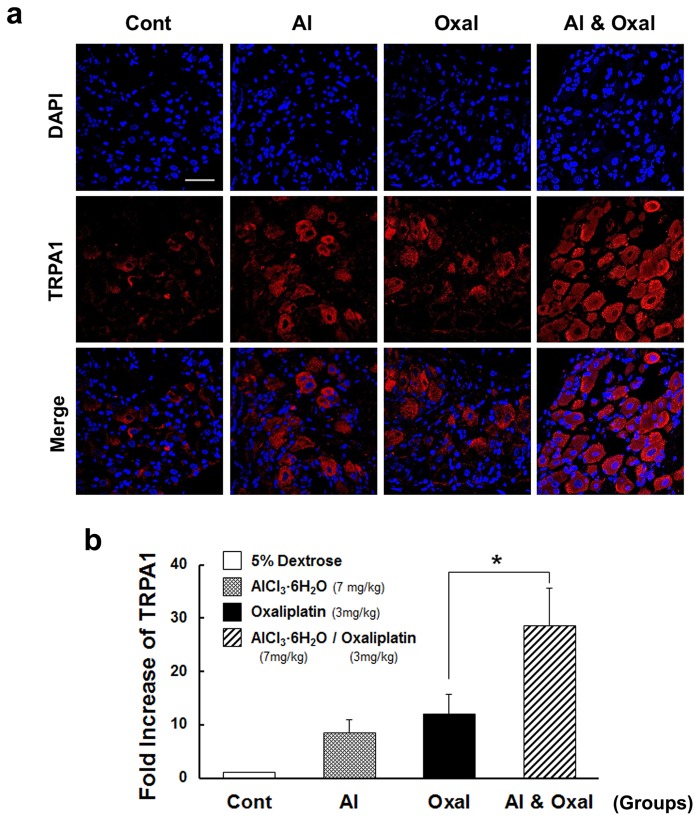
TRPA1 mRNA and protein expression in DRG of mice after combination treatment with oxaliplatin and aluminum chloride. DRG tissues from Cont (5% dextrose), Al (aluminum chloride; AlCl_3_·6H_2_O, 7 mg/kg; equivalent 0.78 mg/kg of elemental Al), Oxal (oxaliplatin, 3 mg/kg), and Al & Oxal (aluminum chloride 7 mg/kg and oxaliplatin 3 mg/kg) groups were harvested at 6 days after the final infusion treatment. (a) Immunofluorescent staining for protein expression was conducted on DRG cryo-sections with anti-TRPA1. Nuclei were stained with DAPI (blue) and visualized by confocal scanning microscopy. The TRPA1 (red) protein level was significantly higher in DRGs from the Al & Oxal groups compared with the Al or Oxal groups. (b) The relative ratio of TRPA1 mRNA measured by quantitative real-time PCR was significantly higher in the DRG from the Al & Oxal compared with that of Al or Oxal. TRPA1 levels are expressed as fold changes after normalizing to 28S RNA. The experiment was conducted in triplicate. Values are expressed as the mean ± SEM (*n* = 10 per group). *p < 0.05 compared with the oxaliplatin group. Scale bar = 40 μm for all panels.

Using quantitative real-time PCR and immunofluorescent staining of DRG tissue, we tested the correlation between behavioral responses to cold allodynia and the expression of TRPA1 protein and mRNA after combinational treatment. Using confocal microscopy, we observed TRPA1 localization in serial sections of DRG tissues after immunofluorescent staining with an anti-TRPA1-antibody. Very little signal was detected in the DRG of the Cont group. Likewise, in the Oxal and Al groups, levels of TRPA1 protein were low. However, in the Al & Oxal group, expression in the lumber DRG was significantly increased relative to the Cont group. Specifically, strong TRPA1 expression (red color) was observed in the cytoplasm, and did not overlap with signals from the nucleus (blue color; Fig 7a). In mice treated with aluminum chloride and oxaliplatin combined (Al & Oxal), TRPA1 mRNA expression was 26-fold higher than in the Cont group, and 2.4-fold higher than in the Oxal group (*p* < 0.05, Fig 7b). The Al group also showed increased TRPA1 expression, though at a lower level than either the Oxal or the Al & Oxal groups. As observed in the previous behavioral results (Fig 3b), TRPA1 protein and mRNA levels in the combinational group also exhibited a synergetic increase (Al & Oxal).

### Effects of treatment with oxaliplatin and aluminum chloride on cell death in the DRG

**Fig 8:**
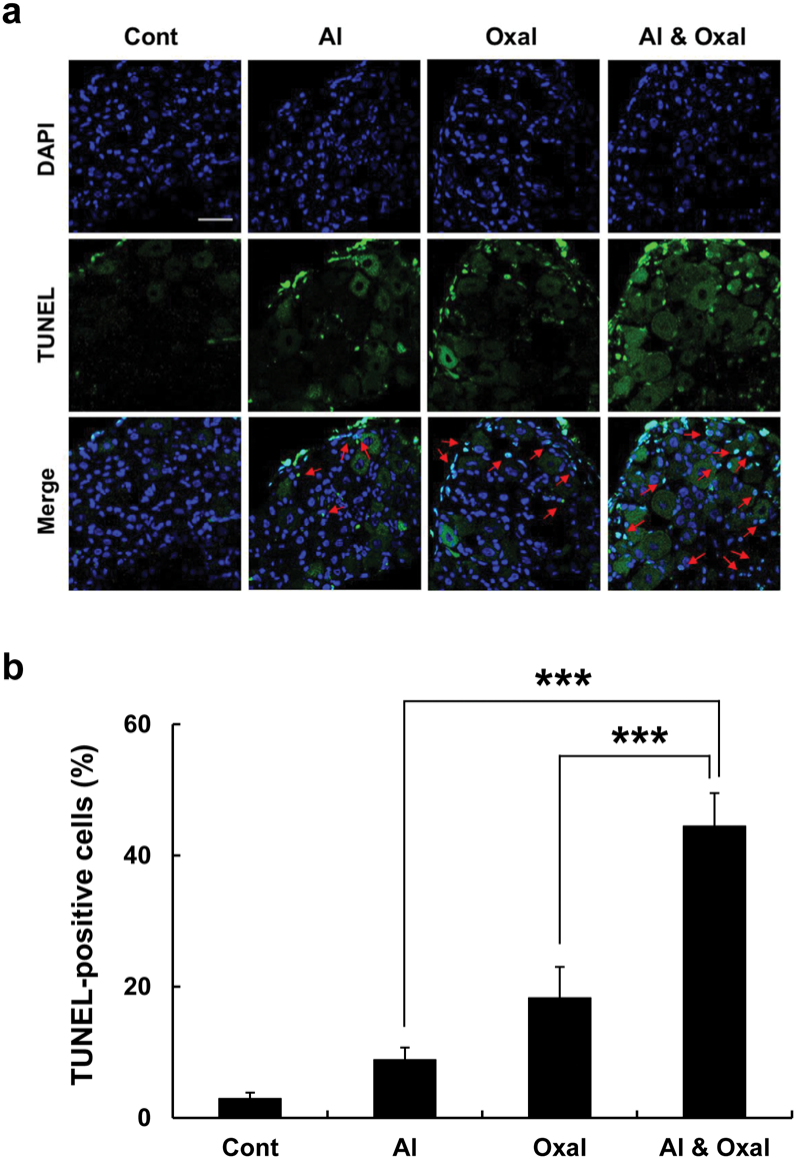
Cell death in DRGs after treatment with oxaliplatin and Al. (a) Cryo-sectioned DRG tissues of Cont (5% dextrose), Al (aluminum chloride, 7 mg/kg; equivalent 0.78 mg/kg of elemental Al), Oxal (oxaliplatin, 3 mg/kg), and Al & Oxal (aluminum chloride 7 mg/kg and oxaliplatin 3 mg/kg) groups were stained using terminal deoxynucleotidyl transferase-mediated dUTP nick end-labeling (TUNEL; green) to evaluate apoptosis. Nuclei were stained with DAPI (blue) and visualized using a confocal scanning microscope. The increase in apoptosis in the DRG from the Al & Oxal group was significant compared to the Oxal and Al groups (red arrows). Scale bar = 40 μm for all panels. (b) Depicting a statistical analysis of TUNEL positive cells. The number of TUNEL positive cells was counted. Values are expressed as the mean ± SEM (*n* = 3 per group). ***p < 0.001 compared with Al group.

To investigate the relationship between cell death and neuropathy caused by oxaliplatin and Al, TUNEL analysis was performed. TUNEL-positive cells were rarely observed in DRG tissue from the Cont group, but were occasionally observed in the Oxal and Al groups. However, DRG cells from animals treated with oxaliplatin and aluminum chloride in combination (Al & Oxal) showed marked increases in TUNEL-positive cells (Fig 8). These data suggested that cell death in the DRG was due to oxaliplatin and/or Al, and may be correlated with neuropathy. In all treatment groups, there was a trend toward an increase of TUNEL-positive cells with Al-, Oxal-, and Al & Oxal group compared to Cont group. The number of TUNEL-positive cells in an Al-, Oxal-, and Al & Oxal group was significantly 3-, 6- and 15-fold more respectively than Cont group (Fig 8b).

## Discussion

In the present study, treatment of mice with oxaliplatin for 4 and 8 weeks induced acute and sub-acute neuropathy, respectively. Testing for cold allodynia using an acetone test showed that peripheral neuropathy induced in these oxaliplatin-treated mice was similar to that described in previous studies [26, 30, 31]. Cold allodynia was also observed in the mice treated with aluminum chloride alone, and pre-treatment with aluminum chloride intensified the oxaliplatin-induced cold allodynia compared to mice treated with oxaliplatin alone. Even without aluminum chloride pre-treatment, oxaliplatin-treated mice accumulated Al in the DRG to levels higher than in mice without any infusion. In DRG tissues from oxaliplatin-treated mice, the expression of TRPA1 was increased, which is consistent with existing characterizations of TRPA1 as a chemosensor molecule [32]. Besides, the TRPA1 expression was more increased in aluminum chloride combinational treatment than oxaliplatin alone. Al toxicity is not common in humans, but disease may develop through prolonged exposure [33–35]. Although the neurotoxicity of metals such as Al [36, 37] is well established, a correlation between Al exposure and peripheral neuropathy has not been determined. Based on our findings, we suggest that accumulation of Al in the body may exacerbate the peripheral neuropathic pain caused by oxaliplatin.

In the present study, oxaliplatin-treated mice exhibited neuropathy in both an acute and sub-acute manner on day 15 and 60 after oxaliplatin infusion, respectively. In particular, sub-acute peripheral neuropathy on day 60 presented as a delayed response to cold stimuli, with a delayed onset. This phenomenon is similar to the clinical symptoms of chronic oxaliplatin-induced neuropathy [4, 38]. The delayed response, representing a kind of numbness, did not correspond to recovery from cold hypersensitivity, because levels of TRPA1 mRNA and protein in the DRG were elevated. Acute cold hypersensitivity is the most common side effect caused by oxaliplatin treatment. However, the cumulative oxaliplatin doses can potentially have the following adverse effects: neurotoxicity, especially irreversible tingling or numbness and intense pain; pulmonary toxicity, especially fibrosis; hepatotoxicity; neutropenia; nausea or vomiting; and diarrhea [39].

Among the many studies seeking to explain the underlying mechanism of peripheral neuropathy, one demonstrated a mechanical change involving the calcium-permeable cation channel TRPA1. According to Nassin *et al*., activation of TRPA1 by glutathione-sensitive molecules, reactive oxygen species, and metabolic byproducts of Pt-based drugs contributes to mechanical and cold hypersensitivity [6]. That study also showed that TRPA1-deficient mice fail to develop mechanical and cold hypersensitivity after oxaliplatin treatment. In another study, acute cold hypersensitivity after oxaliplatin treatment was shown to be caused by enhanced responsiveness of TRPA1, but not TRPM8 [40] or TRPV1 [41, 42]. TRPA1 play a role in cold sensing but in other study, as another cold transducer, the transient receptor potential melastatin 8 (TRPM8) also plays a role on oxaliplatin-induced peripheral neurotoxicity [43]. There was no report about direct activation of TRPM8 channels by oxaliplatin but TRPM8 might play a role in cold sensing in oxaliplatin-induced neuropathy by the observation with TRPM8 knock out animals [40]. Therefore the major role between TRPM8 and TRPA1 for cold sensing in oxaliplatin-induced neuropathy is not clearly identified and further studies are needed [44]. On the other hand, some studies have shown an association between thermal hyperalgesia or allodynia evoked by Pt-based drugs and the transient receptor potential vanilloid 1 (TRPV1) [45]. Other studies, including our own, have not identified changes in response to thermal stimulation following treatment with Pt-based drugs [46]. In the present study, behavioral test results for the peripheral neuropathy induced by infusion of aluminum chloride and oxaliplatin, each alone or both in combination, correlated with the activation of TRPA1 mRNA and protein expression. To more investigate the involvement of other TRP channels in oxaliplatin induced peripheral neuropathy, further studies should be performed.

Daily human Al consumption comes from edible plants (notably tea and certain herbs) that accumulate minerals from soil; additives to prepared foods such as pickles, processed cheese and baking powder; medicines such as antacids; and water from wells in some geological formations. Neurotoxicity resulting from Al exposure has been widely reported [18, 47, 48]. Kumar *et al*. demonstrated that chronic exposure to Al leads to disruption of microtubules and neurofilament hyperphosphorylation in rodents, thus revealing a potential mechanism of neurotoxicity [49]. Al has also been proposed to induce neurotoxicity by altering oxidation-reduction status and inhibiting antioxidant enzymes such as superoxide dismutase (SOD), catalase, and glutathione peroxidase (GSH-Px) in the central nervous system. Assays of thiobarbituric acid-reactive substances (TBARS) in nervous tissues support this type of mechanism [50]. The Al-induced peripheral neuropathy that we observed in mice was similar to neuropathies induced by oxaliplatin, except in the case of a withdrawal response measured in the early phase compared with oxaliplatin alone. Expression of TRPA1 mRNA and protein was also activated in the Al-induced neuropathy model. However, the oxaliplatin group pre-treated with aluminum chloride had the same onset time of neuropathy as aluminum chloride alone, but also more pronounced cold-sensitivity in the late phase than the group treated with oxaliplatin alone. Interestingly, we found that Al accumulation in the DRG was greater in mice treated with aluminum chloride and oxaliplatin in combination than in those treated with aluminum chloride alone, whereas the Pt level was similar to oxaliplatin alone. This phenomenon was also observed in mice treated with oxaliplatin alone, as compared with mice that received infusion of 5% dextrose. These findings were correlated with the results of behavioral testing for cold allodynia. Thus, oxaliplatin seems to progressively increase Al accumulation in DRG with cumulative doses, and cold hyperalgesia may increase incrementally with Al accumulation.

Oxalate, the second metabolite of oxaliplatin, chelates metallic cations such as Ca^2+^ and Mg^2+^. In contrast, Al is a metalloid (semiconductor) element with high ionization potential in water that may form an insoluble black precipitate of aluminum oxalate [51]. Hence, it is possible that oxalate may promote Al accumulation through chelation and inhibition of Al excretion. Protocols for preparation of oxaliplatin prior to clinical use include a precaution against use of instruments containing Al to administer the drug, because these may cause degradation of Pt compounds [52]. This interaction between Pt and Al *ex vivo* supports the occurrence of a parallel interaction *in vivo* that may affect the antitumor efficacy of oxaliplatin.

A plausible component of Al-induced peripheral neuropathy was revealed by our finding that apoptosis increased in the DRG after oxaliplatin/aluminum chloride treatment compared with oxaliplatin alone. Previous research also shows that Al induces apoptotic and necrotic cell death, which results in shrinkage of cell bodies and degeneration of cellular processes [53]. Oxaliplatin is reported to cause damage to cell bodies, alterations in the nuclei, and atrophy of a subpopulation of DRG neurons [54, 55]. Therefore, the prominent signals observed in both the nucleus and the cytoplasm may represent coincidental apoptotic and necrotic events in DRG tissue after co-treatment with oxaliplatin and aluminum chloride [29]. Based on the results of our experiments, we showed that oxaliplatin-induced neuropathy was induced by TRPA1 activated by aluminum accumulation but we did not show the direct evidence about aluminum accumulation in DRG induced by oxaliplatin. Therefore, to prove a causal relationship between TRPA1 expression induced by oxaliplatin treatment and its coincidence with the time of aluminum accumulation, TRPA1 inhibitor (HC030031, ChemBridge, San Diego, CA) challenging experiment need to be examined in vivo system [56–58]. This experiment may open the window for a direct evidence to investigate the correlation among the role of TRPA1, aluminum accumulation and oxaliplatin-induced neuropathy.

Some metallic elements are proposed to influence cancer development and progression. In comparing human lung tumor tissue and normal lung tissue, significantly higher concentrations of six elements (Al, Cr, Cu, Fe, Na, and Zn) are detected in tumor tissue than in normal tissue [59]. These elements may potentially affect diverse physiological processes directly or indirectly related to cancer development [60]. Interestingly, one may surmise based on these findings that metal accumulation may increase with cancer progression. In our murine induced-tumor study, we found significant Al accumulation in tumor tissues, and these levels increased further after chemo-infusion. This finding suggests that some metals stored in tumors and Pt accumulation through chemotherapy may increase associated neurotoxicity. However, we could not use this tumor model for further investigation of chemo-induced peripheral neuropathy because the growth of tumors directly caused hypesthesia before chemotherapy (data not shown).

Intensive research has sought to reveal the mechanisms of chemo-induced peripheral neuropathy, and to identify and test medications that alleviate this effect. However, these medications vary in effectiveness between patients and typically prove ineffective over long-term exposure to chemotherapy. In the present study, we demonstrated that Al accumulation augments the peripheral neuropathy induced by oxaliplatin through activation of TRPA1 and induction of cell death in the DRG. In the present study, we demonstrated for the first time *in vivo* that Al accumulation augments the peripheral neuropathy induced by oxaliplatin through activation of TRPA1 and induction of cell death in the DRG. Based on these findings, we propose that oxaliplatin-induced peripheral neuropathy may be alleviated by agents that chelate Al. However, the relationship between elemental accumulation in tumors and biological activities of chemotherapeutic drugs awaits further investigation.
